# Neutrophil-guided dosing of anthracycline–cyclophosphamide-containing chemotherapy in patients with breast cancer: a feasibility study

**DOI:** 10.1007/s12032-015-0550-x

**Published:** 2015-03-13

**Authors:** Jan C. Drooger, Johanna M. van Pelt-Sprangers, Corry Leunis, Agnes Jager, Felix E. de Jongh

**Affiliations:** 1 Department of Internal Medicine, Ikazia Hospital, PO Box 5009, 3008 AA Rotterdam, The Netherlands; 2 Department of Medical Oncology, Erasmus MC Cancer Institute and Cancer Genomics Netherlands, PO Box 5201, 3008 AE Rotterdam, The Netherlands; 3 Department of Internal Medicine, Erasmus MC, PO Box 2040, 3000 CA Rotterdam, The Netherlands

**Keywords:** Neutrophil-guided dose escalation, Chemotherapy, Breast cancer, Anthracyclines, Cyclophosphamide

## Abstract

The aim of this study was to investigate whether neutrophil-guided dose escalation of anthracycline–cyclophosphamide-containing chemotherapy (ACC) for breast cancer is feasible, in order to optimize outcome. Breast cancer patients planned for 3-weekly ACC were enrolled in this study. The first treatment cycle was administered in a standard BSA-adjusted dose. The absolute neutrophil count was measured at baseline and at day 8, 11 and 15 after administration of ACC. For patients with none or mild (CTC grade 0–2) neutropenia and no other dose-limiting toxicity, we performed a 10–25 % dose escalation of the second cycle with the opportunity to a further 10–25 % dose escalation of the third cycle. Thirty patients were treated in the adjuvant setting with either FE100C (*n* = 23) or AC (*n* = 4), or in the palliative setting with FAC (*n* = 3). Two out of 23 patients (9 %) treated with FEC did not develop grade 3–4 neutropenia after the first treatment cycle. Dose escalation was performed in these two patients (30 % in one and 15 % in the other patient). During dose escalation, there were no complications like febrile neutropenia. No patients treated with FAC or AC could be escalated, since all of them developed grade 3–4 neutropenia. We conclude that asymptomatic grade 3–4 neutropenia is likely to be achieved in the majority of patients with breast cancer treated with ACC according to presently advocated BSA-based dose levels. Escalation of currently advocated ACC doses without G-CSF, with a target of grade 3–4 neutropenia, is feasible, but only possible in a small proportion of patients. EudraCT 2010-020309-33.

## Introduction

Both anthracyclines and cyclophosphamide are highly effective drugs in the treatment of breast cancer [1, 2]. According to international guidelines, anthracycline–cyclophosphamide-containing chemotherapy (ACC) is part of (neo) adjuvant treatment schedules for early stage or locally advanced breast cancer [3]. Furthermore, in the setting of metastatic disease, ACC is often used as palliative treatment [4, 5].

Although highly effective, not all patients benefit from ACC. The cumulative dose of anthracyclines administrated is important. From randomized controlled trials, it is clear that higher ‘standard dose’ of anthracyclines for early breast cancer improves patient survival compared to lower ‘standard dose’ [6]. On the other hand, a reason for differences in efficacy among patients who have had a similar dose of anthracyclines administered could be the large inter-individual (between patients) as well as the intra-individual (within patients) variability in pharmacokinetic (PK) parameters [7]. Interestingly, hematological toxicity is strongly associated with the absolute dose of anthracycline and might be useful as a surrogate measure of the anthracycline dose [6]. In accordance, some retrospective studies indeed have shown that breast cancer patients given adjuvant chemotherapy but not attaining at least moderate hematological toxicity have a worse prognosis compared to those with more toxicity [8–11].

The current standard of dosing ACC is guided by body surface area (BSA) with an a posteriori dose reduction of all component drugs in case of excessive toxicity (e.g., febrile neutropenia). Dose escalation among patients without toxicity is, however no standard of care. The administration of an inappropriately low dose of chemotherapy is therefore not recognized, leaving patients that might benefit from an increased dose unidentified. The percentage of breast cancer patients receiving a suboptimal dose of ACC is unknown, as well as the amount of under-dosing in these individuals.

In the present study, we addressed the feasibility of a simple tool for neutrophil-guided dose adaptation of ACC (without primary G-CSF support), among female breast cancer patients treated with ACC for either palliative or curative intention. The aim was to reach nadir absolute neutrophil count (ANC) of ≤1.0 × 10e9/L with recovery to ≥1.5 × 10e9/L at the time of the planned next treatment cycle, without excessive hematological or non-hematological toxicity. In case successful dose escalation is possible in a substantial number of patients, this method is valid and should be further developed and refined to be ultimately tested on treatment efficacy in a prospective randomized trial of neutrophil-guided versus standard BSA-adjusted dosing.

## Patients and methods

### Participants

Chemotherapy-naive female breast cancer patients aged ≥18 years and planned for treatment with at least three cycles of ACC were identified at the Department of Medical Oncology, Ikazia Hospital, Rotterdam, The Netherlands. Both patients treated with curative as well as patients treated with palliative intention were eligible. Patients with the following chemotherapy regimens were eligible: FEC (fluorouracil 500 mg/m^2^, epirubicin 100 mg/m^2^, cyclophosphamide 500 mg/m^2^), AC (doxorubicin 60 mg/m^2^, cyclophosphamide 600 mg/m^2^) or FAC (fluorouracil 500 mg/m^2^, doxorubicin 50 mg/m^2^, cyclophosphamide 500 mg/m^2^). Additionally, patients should have a WHO performance status 0–1, life expectancy >3 months, adequate peripheral blood cell counts (leukocytes ≥4.0 × 10e9/L and ANC ≥2.0 × 10e9/L and platelet count ≥150 × 10e9/L), adequate renal function (defined as normal serum creatinine concentration and/or estimated creatinine clearance ≥60 mL/min), adequate liver function [defined as normal serum bilirubin concentration (≤17 μmol/L) and serum ASAT and ALAT ≤3 times the upper limit of normal (≤5 times the upper limit of normal in case of hepatic metastases)], normal serum albumin concentration (35–50 g/L) and given written informed consent. Women were excluded from participation if they had been treated with chemotherapy previously, were unable to consent with weekly follow-up for blood cell counts and toxicity assessment, had symptomatic brain metastasis, had a history of cardiac dysfunction, had uncontrolled arterial hypertension (blood pressure systolic ≥180 mmHg and/or diastolic ≥110 mmHg) and/or unstable angina pectoris. Ethical approval for this study was obtained through the Institutional Review Boards, and all women signed the informed consent. The study was conducted in full accordance with the principles of the Declaration of Helsinki and local regulations. The trial adhered to the guidelines for good clinical practice and the European Union Clinical Trial Directive.

### Study design

**Fig. 1:**
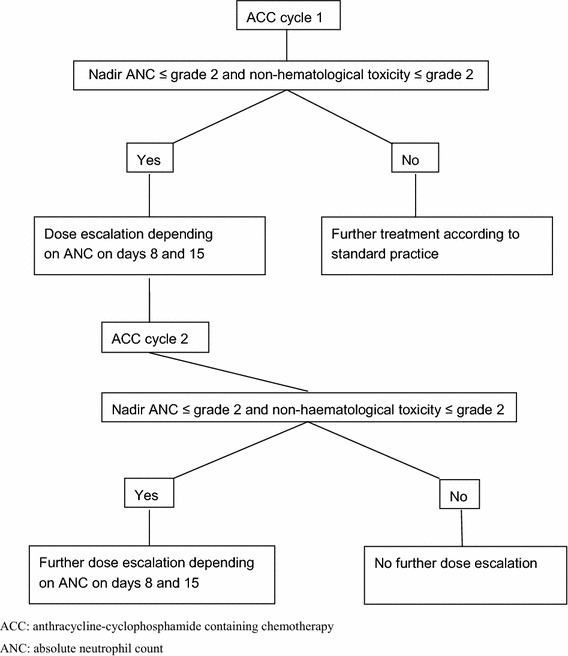
Study design. *ACC* anthracycline–cyclophosphamide-containing chemotherapy, *ANC* absolute neutrophil count

This study was a prospective single-center feasibility study. The first treatment cycle was given using standard BSA-adjusted dosing. Following the administration of ACC, ANC was evaluated in peripheral venous blood samples obtained at days 8 (±1), 11 (±1) and 15 (±1), day 1 being the day of chemotherapy administration. Hematological and non-hematological toxicities were assessed weekly according to the common toxicity criteria (CTC), version 3. Subsequent cycles of ACC were given at 3-week intervals provided that the patient had sufficiently recovered from hematological and non-hematological toxicity. Sufficient recovery of hematological toxicity was defined as an ANC of ≥1.5 × 10e9/L and a platelet count of ≥100 × 10e9/L, whereas sufficient recovery of non-hematological toxicity was defined as CTC grade ≤1 (with the exception of alopecia). In patients with nadir ANC ≥1.0 × 10e9/L and maximum non-hematological toxicity CTC grade ≤2 during the first cycle of ACC, the dose of cyclophosphamide and the anthracycline (doxorubicin or epirubicin) was increased with 10, 15 or 25 % according to a predefined schedule based on neutrophil count on day 8 and day 15. In patients treated with chemotherapy schedules including fluorouracil (FAC or FEC), the dose of fluorouracil was not escalated due to its negligible contribution to hematological toxicity in these combination regimens [7]. Patients undergoing dose escalation of the second cycle of ACC were candidates for a further (and final) dose escalation of the third cycle of ACC following the same principles and according to the same predefined schedule. Patients experiencing excessive toxicity (i.e., febrile neutropenia, symptomatic thrombocytopenia and/or grade 3–4 non-hematological toxicity with the exception of nausea and vomiting) without previous dose escalation were treated according to standard clinical practice. In case of excessive toxicity after dose escalation, patients had to be retreated with standard BSA-adjusted dose during subsequent treatment cycles. Finally, all patients treated with ≥4 cycles of ACC received standard BSA-adjusted dosing from the fourth cycle onward. The study design is also outlined in Fig. 1.

### Statistical analysis

This study was designed as a pilot feasibility study. Therefore, a useful sample size calculation was not appropriate. We aimed to enter 30 patients. Successful dose escalation of chemotherapy was our primary goal and was rather arbitrarily defined as a ≥15 % increase in anthracycline/cyclophosphamide dose without excessive hematological (febrile neutropenia, grade 3–4 thrombocytopenia) or non-hematological (grade 3–4) toxicity. We stated that if successful dose escalation was possible in a significant proportion of patients (at least three out of 30 patients), our experimental method of neutrophil-guided dose escalation could be feasible in daily clinical practice and should be further developed and refined to be ultimately tested on treatment efficacy in a prospective randomized trial of neutrophil-guided versus standard BSA-adjusted dosing. If successful dose escalation turned out to be possible in less than three out of 30 patients, it is unlikely that this method of dose escalation will have significant impact on treatment efficacy, and this method should not be further explored.

Furthermore inter-individual variation in ANC after administration of the chemotherapy was assessed as coefficient of variation (CV) for nadir ANC and for cumulative neutrophil count [expressed as the sum of CTC grades of neutropenia (0–4) on day 8, 11 and 15], addressing the duration of neutropenia.

## Results

A total of 30 patients were entered in this study between November 2010 and December 2013. Baseline characteristics are outlined in Table 1. Median age was 55 years (range 37–74 years). The majority of the patients were treated for early breast cancer with either FEC (77 %) or AC (13 %). Three patients (10 %) were treated with first-line palliative chemotherapy in the form of FAC.

**Table 1:** Baseline characteristics

Median age [years (range)]	55 (36–74)
Chemotherapy regime [*n* (%)]	
FEC	23 (77)
FAC	3 (10)
AC	4 (13)
Tumor stage [*n* (%)]	
Early	27 (90)
Metastatic	3 (10)
WHO performance score [*n* (%)]	
WHO 0	25 (83)
WHO 1	5 (17)
Median height [cm (range)]	170 (155–184)
Median weight [kg (range)]	75 (53–100)
Median body surface are [m^2^ (range)]	1.9 (1.5–2.1)

**Fig. 2:**
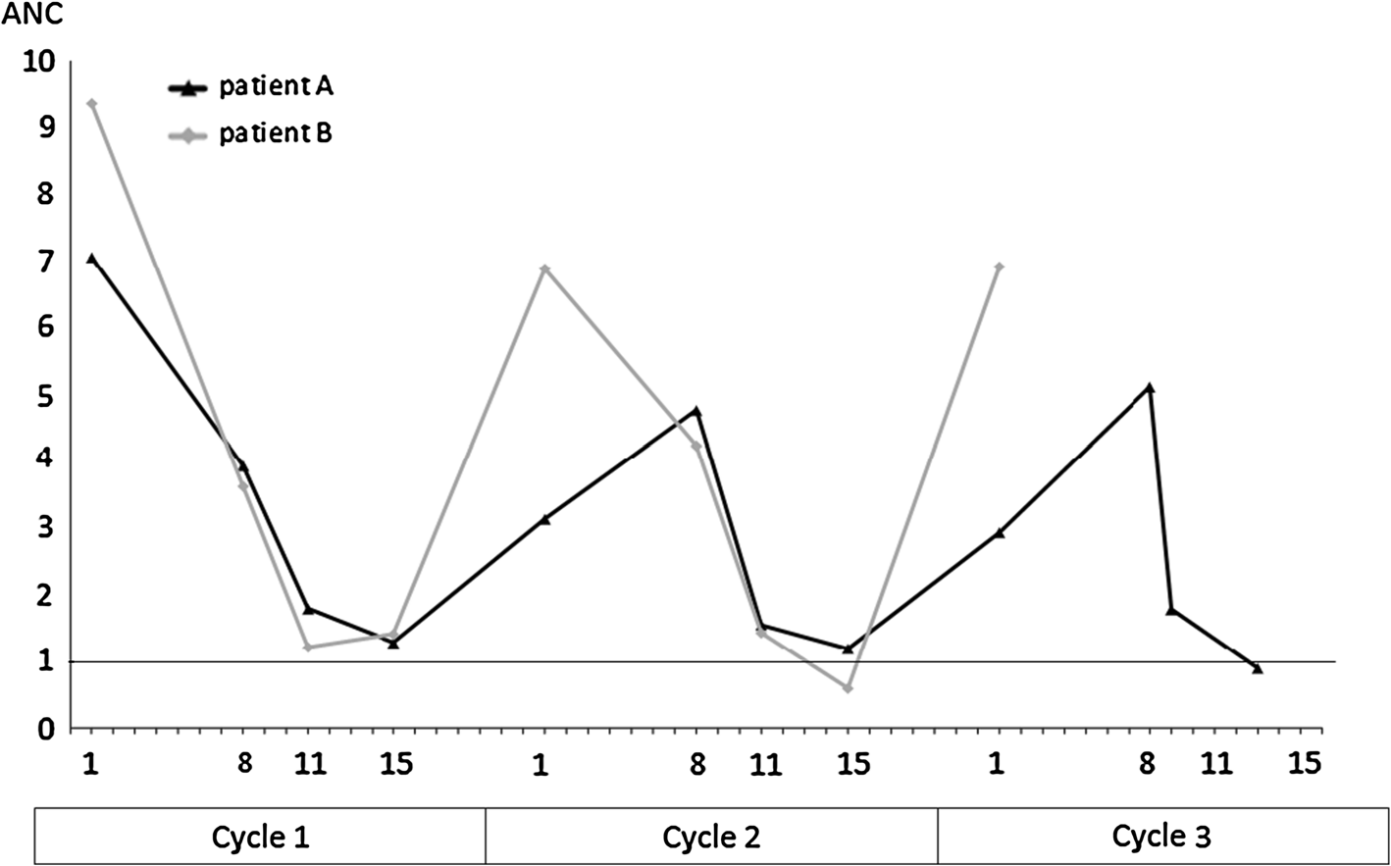
Absolute neutrophil counts over time in the two escalated patients

Dose escalation was feasible in two patients. Both patients were treated with FEC for early breast cancer. So 2/23 (9 %) of patients treated with FEC could be escalated, while no patients treated with FAC or AC could be escalated. Both of the escalated patients developed only grade 2 neutropenia (ANC 1.00–1.49 × 10e9/L) at day 15 of the first cycle and were escalated with 15 % during the second cycle. One of these patients reached grade 3 neutropenia (ANC 0.50–0.99 × 10e9/L) in the second cycle, and no further escalation was performed. The other patient developed only grade 2 neutropenia after the second cycle and was further escalated with another 15 % in cycle 3 (Fig. 2; Table 2). During dose escalation, there were no complications like febrile neutropenia, grade 3–4 thrombocytopenia or increase in non-hematological toxicity. There were no relevant differences in baseline characteristics between escalated and non-escalated patients (Table 2).

**Table 2:** Details of escalated patients

	Patient A	Patient B
Escalations	15 % in first cycle	15 % in first cycle
	15 % in second cycle	No further escalation in second cycle
Age (years)	47	55
Chemotherapy regime	FEC	FEC
Body surface are (m^2^)	1.9	1.8
Weight (kg)	75	75
Cycle 1 ANC baseline	7.05	9.35
Cycle 1 ANC nadir	1.28	1.21
Cycle 1 ANC nadir (day reached)	15	11
Cycle 2 ANC baseline (×10e9/L)	3.13	6.88
Cycle 2 ANC nadir (×10e9/L)	1.19	0.59
Cycle 2 ANC nadir, day reached	15	15

*ANC* absolute neutrophil count

For the whole group of patients, mean ANC at baseline was 5.32 × 10e9/L (range 2.66–10.52), on day 8 was 3.72 × 10e9/L (range 2.02–9.60), on day 11 was 0.80 × 10e9/L (range 0.05–2.11) and on day 15 was 0.53 × 10e9/L (range 0.03–3.94). Nadir mean was 0.41 × 10e9/L (range 0.03–1.28) and was reached on day 11 in seven patients and on day 15 in 23 patients. Coefficient of variation (CV) for mean ANC nadir was 0.77. Grade 3 neutropenia (ANC 0.50–0.99 × 10e9/L) or grade 4 neutropenia (ANC <0.50 × 10e9/L) after the first treatment cycle was observed in 28 of the 30 patients. Two of them had febrile neutropenia and were hospitalized. All patients had recovery of ANC to ≥1.5 × 10e9/L at the time of the planned next treatment cycle. Duration of neutropenia expressed as mean cumulative neutrophil count (the sum of CTC grades of neutropenia on day 8, 11 and 15) was 6.4 with a CV of 0.24 (Table 3).

**Table 3:** Laboratory values, first cycle

Mean ANC	
Baseline [×10e9/L (range)]	5.32 (2.66–10.52)
Day 8 [×10e9/L (range)]	3.72 (2.02–9.60)
Day 11 [×10e9/L (range)]	0.80 (0.05–2.11)
Day 15 [×10e9/L (range)]	0.53 (0.03–3.94)
Mean ANC nadir [×10e9/L (range)]	0.41 (0.03–1.28)
Coefficient of variation (CV)	0.77
ANC nadir [*N* (%)]	
Day 8	0 (0)
Day 11	7 (23)
Day 15	23 (77)
Neutropenia nadir [*N* (%)]	
CTC grade 0	0 (0)
CTC grade 1	0 (0)
CTC grade 2	2 (7)
CTC grade 3	8 (27)
CTC grade 4	20 (67)
Mean cumulative neutrophil count^a^	6.4
Coefficient of variation	0.24
Febrile neutropenia [*N* (%)]	2 (7)

^a^Expressed as the sum of CTC grades of neutropenia (0–4) on day 8, 11 and 15

Incidence of grade 3–4 neutropenia was lower after the second and third cycle of chemotherapy compared to after the first cycle (proportion of grade 3–4 neutropenia in cycle one, two and three, respectively, 94, 77 and 75 %, nonsignificant, Table 4). In these figures, patients who underwent dose escalation or used secondary G-CSF prophylaxis were excluded.

**Table 4:** ANC nadir in first three cycles of ACC

	Cycle 1 (*n* = 30)	Cycle 2 (*n* = 21^a^)	Cycle 3 (*n* = 16^b^)
Mean ANC nadir [×10e9/L (range)]	0.41 (0.03–1.28)	0.66 (0.09–1.50)	0.63 (0.06–1.57)
ANC nadir [*N* (%)]			
Day 8	0 (0)	0 (0)	0 (0)
Day 11	7 (23)	2 (10)	5 (31)
Day 15	23 (77)	19 (90)	11 (69)
Neutropenia nadir [*N* (%)]			
CTC grade 0	0 (0)	0 (0)	0
CTC grade 1	0 (0)	0 (0)	1 (6)
CTC grade 2	2 (7)	5 (24)	3 (19)
CTC grade 3	8 (27)	10 (48)	4 (25)
CTC grade 4	20 (67)	6 (29)	8 (50)

*ANC* absolute neutrophil count

^a^Exclusion of escalated patients (*n* = 2), patients with febrile neutropenia after the first cycle (*n* = 2) and patients with missing data (*n* = 5). Besides the escalated patients, there were no patients with dose alterations in the second cycle

^b^Exclusion of escalated patients (*n* = 2), patients with febrile neutropenia after the first cycle (*n* = 2) and patients with missing data (*n* = 10). There were no patients with febrile neutropenia after the second cycle. Besides the escalated patients, there were no patients with dose alterations in the third cycle

## Discussion

The ANCHOR study was designed based on the improved survival found in a number of retrospective studies among patients treated with adjuvant chemotherapy for early breast cancer, who achieved a higher degree of hematological toxicity [6, 9–11]. In this pilot feasibility study among breast cancer patients treated with currently advocated doses of ACC, neutrophil-guided dose escalation was feasible. Dose escalation was possible in two out of 23 patients (9 %) treated with FEC, while dose escalation was possible in none of the patients treated with FAC or AC. Asymptomatic grade 3–4 neutropenia was achieved in the majority of patients after the first cycle of ACC. It seems therefore not useful to proceed with a large randomized controlled trial on neutrophil-guided dose escalation among patients with currently advocated doses of ACC.

Previously, two other studies have also investigated the feasibility of dose escalation of ACC, although they are hardly comparable with our current study. In the first study of tailored fluorouracil, epirubicin and cyclophosphamide (FEC) with primary granulocyte colony-stimulating factor (G-CSF) support, the dose of epirubicin and cyclophosphamide could be escalated by 50 % or more in more than half of the patients. Starting dose in this study was fluorouracil 600 mg/m^2^, epirubicin 75 mg/m^2^ and cyclophosphamide 900 mg/m^2^. Treatment with nine cycles of tailored FEC with G-CSF support (median cumulative dose of epirubicin was 780 mg/m^2^) was, however, associated with an increased risk of acute myeloid leukemia and myelodysplastic syndrome. There were also more cardiac side effects in the tailored FEC group. Tailored FEC with G-CSF support can therefore not be advocated for clinical practice [12]. In our study, the two patients in whom escalation of ACC was feasible had a cumulative dose of epirubicin of 347 and 330 mg/m^2^, respectively. In the second study by Edlund et al., the study design was comparable with our study; however, the ‘standard’ dose of epirubicin used in this study was substantially lower than in our study (60 mg/m^2^ vs 100 mg/m^2^, respectively). In this study (*n* = 1535), patients who did not reach leukopenia CTC grade 3 or 4 after a first cycle of standard FEC (in this study fluorouracil 600 mg/m^2^, epirubicin 60 mg/m^2^ and cyclophosphamide 600 mg/m^2^) were randomized to a total of six courses of standard dosed FE(60)C (*n* = 526) or a total of six cycles of FEC with doses tailored to achieve grade 3 leukopenia (*n* = 521). The relative dose intensity (defined as the given dose delivered in the originally expected time/the expected dose in the expected time) was increased by a factor of 1.31. Median cumulative dose of epirubicin in the tailored dose group was 520 mg/m^2^. There was no excess of acute non-hematological toxicity [13].

It is important to mention that both these studies used lower ‘standard’ doses of epirubicin compared to our study (75 and 60 mg/m^2^, respectively). It can be concluded based on these and our study that neutrophil-guided dose escalation might be feasible in older regimens with lower ‘standard dose’ of epirubicin. With currently advocated doses (epirubicin 100 mg/m^2^ and doxorubicin 50–60 mg/m^2^), it is, however, not feasible to escalate a relevant proportion of patients.

Interestingly, the two patients, who were escalated, were both treated with epirubicin, while none of the patients treated with doxorubicin could be escalated. This might be due to chance. A real difference in hematological toxicity between these two anthracycline can, however, not be excluded. When taking only the patients treated with epirubicin into account, dose escalation was possible in 9 % of patients, not reaching the predefined 10 % which was considered worthwhile enough for further exploration.

Furthermore, a trend was seen in a decreased proportion of patient with grade 3–4 neutropenia over the subsequent cycles. In our study, dose escalation was only permitted when no grade 3–4 neutropenia was seen after cycle 1. When we also had allowed patients to escalate based on ANC nadir after the second cycle, five more patients could have been escalated in the third cycle. One of the currently advocated (neo)adjuvant chemotherapy regimens consists of three cycles FEC (5FU 500; epiriubicin 100 and cyclophosphamide 500 mg/m^2^), followed by three cycles docetaxel 100 mg/m^2^. In this regimen, cumulative anthracycline dose is relatively low, with a low risk of cardiotoxicity [14]. Since only three cycles of ACC are given, it is of utmost importance to dose these cycles as high as possible without unacceptable side effects. Further research should therefore focus mainly on patients treated in (neo)adjuvant setting and allow escalation also in subsequent cycles.

For most classical anticancer drugs, BSA-guided dosing is still standard practice in clinical oncology. BSA-based dosing of chemotherapy has largely resulted from its use in the extrapolation of drug doses used in experimental animals to those considered safe as starting doses for phase I clinical trials. However, a proper scientific rationale for BSA-based dosing of anticancer drugs in human adult cancer patients is lacking [15–17]. Furthermore, the use of BSA does not reduce inter-individual variability in pharmacokinetic parameters for the majority of investigated anticancer drugs [18]. For irinotecan, it has been shown that flat-fixed dosing does not result in increased pharmacokinetic/pharmacodynamic variability and could be safely used [19]. Furthermore, for carboplatin, glomerular filtration rate-adjusted dosing has been widely accepted as standard [20]. Although knowledge on pharmacogenetics has rapidly been expanding, this had not led to many practically applicable dosing algorithms for classical anticancer drugs, while exposure to chemotherapy is influenced by many other interacting factors [21, 22]. For fluoropyrimidines, it has been suggested to adjust the dose based on dihydropyrimidine dehydrogenase (DPYD) genotype tests [23]. The method of dose adjustment guided by plasma drug concentrations (therapeutic drug monitoring, TDM) has not been used as standard practice, which is largely due to the obscure relationship between plasma drug concentrations and treatment effects [24, 25]. However, for the vast majority of classical anticancer agents, BSA-guided dosing remains still standard practice. For most (oral) targeted agents, flat-fixed dosing and a posteriori dose reduction in case of severe toxicity is a standard practice. For these agents, the relation between dose and outcome (both efficacy and safety) is even less clear, compared to classical chemotherapy, due to both differences in molecular characteristics of the tumor as well as in environmental and genetic factors.

## Conclusion

In conclusion, inter-individual variability in hematological toxicity with currently advocated doses of ACC in breast cancer patients is limited. Escalation of currently advocated ACC doses without G-CSF, with a target of grade 3–4 neutropenia, is feasible, but only possible in a relatively small proportion of patients. Since no other dosing algorithms are available for ACC, BSA-guided dosing remains standard practice at this moment.
